# Chemical reaction networks based on conjugate additions on β′-substituted Michael acceptors

**DOI:** 10.1039/d3cc02126b

**Published:** 2023-07-26

**Authors:** Benjamin Spitzbarth, Rienk Eelkema

**Affiliations:** a Department of Chemical Engineering, Delft University of Technology Van der Maasweg 9 2629 HZ Delft The Netherlands R.Eelkema@tudelft.nl

## Abstract

Over the last few decades, the study of more complex, chemical systems closer to those found in nature, and the interactions within those systems, has grown immensely. Despite great efforts, the need for new, versatile, and robust chemistry to apply in CRNs remains. In this Feature Article, we give a brief overview over previous developments in the field of systems chemistry and how β′-substituted Michael acceptors (MAs) can be a great addition to the systems chemist's toolbox. We illustrate their versatility by showcasing a range of examples of applying β′-substituted MAs in CRNs, both as chemical signals and as substrates, to open up the path to many applications ranging from responsive materials, to pathway control in CRNs, drug delivery, analyte detection, and beyond.

## Abstract 2

The diverse reactivity of β′-substituted Michael acceptors make them a perfect fit for the application in Chemical Reaction Networks (CRNs). We show how these CRNs can find use in material science, signal amplification, and beyond.

## Graphical abstract

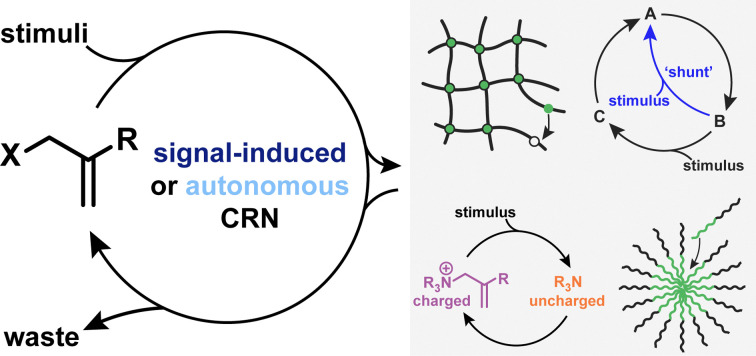

## Introduction

### Systems chemistry: chemical toolbox to understand and mimic Nature

**Fig. 1:**
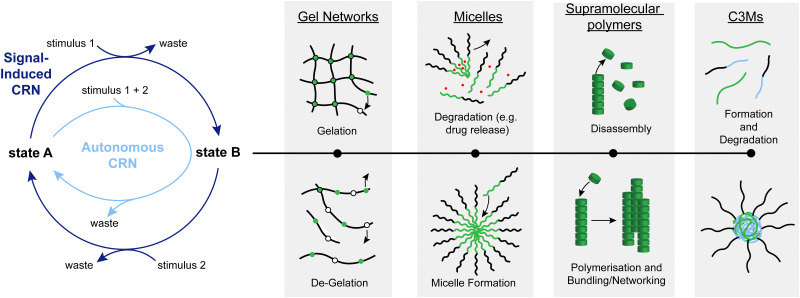
General scheme of a CRN. Under the influence of a stimulus, state A is transformed into state B which can perform a function, with some examples depicted on the right. A second stimulus can recover state A from state B.

Systems chemistry initially evolved out of a desire to develop and study more complex chemical systems to bridge the large gap between traditional chemical processes and biochemical reaction networks.^1^ As a result, over the last couple of decades, a wide range of artificial chemical reaction networks (CRNs) have been developed.^2^ They have many functions and can impart interesting properties such as transient formation, cargo release, or autonomous behaviour on other systems such as fuelled, transient materials (*e.g.* supramolecular assemblies,^3,4^ micelles,^5–8^ complex coacervate core micelles (C3Ms),^9–12^ crosslinked hydrogels,^13–15^ and sensors (*e.g.* pH switches^16–19^ and light-controlled switches^20–22^),^23^ among others. Such CRNs typically consist of a cycle of chemical reactions, starting from and ending with the same chemical species (Fig. 1, left). In autonomous cycles, a stimulus (such as chemical fuel(s), changes in pH, or irradiation) is used to transform the starting material into a new species, which typically leads to an out-of-equilibrium situation (*i.e.* a situation in which the thermodynamic minimum of the system has not been established yet), where the back reaction recovers the starting material (Fig. 1, light blue). The driving force of the cycle maintains the system in an out-of-equilibrium state until the stimulus is used up or not applied anymore. In signal-induced cycles, a reagent is added that transforms state A into state B (Fig. 1, dark blue). State B will then only be transformed back to state A when a second reagent (a signal) to initiate the back-reaction is added. Whether cycles can be run autonomously or in a signal-induced manner depends on several factors such as orthogonality of all stimuli and desired lifetime of state B.

**Scheme 1:**
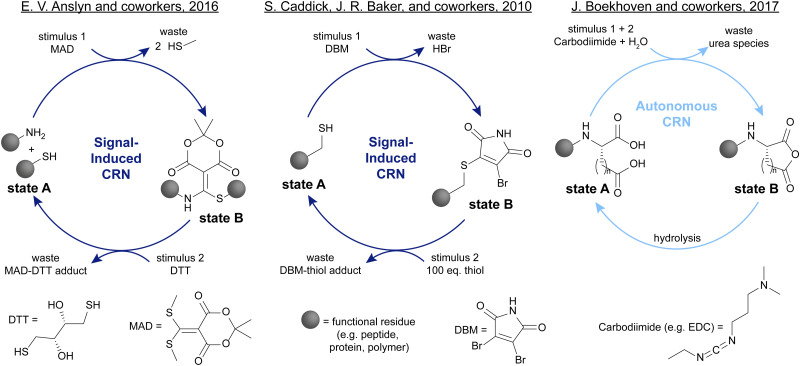
Recent examples of signal-induced and autonomous CRNs. Left: Anslyn and coworkers showed that Meldrum's acid derivative (MAD) can be used to selectively capture one thiol and one amine moiety which can be released again upon addition of DTT.^24^ Centre: Caddick, Baker, and coworkers demonstrated the reversible functionalisation of biologically relevant thiols with dibromomaleimides (DBM).^26^ Right: Boekhoven and coworkers introduced the transient formation of anhydrides from acids with carbodiimides such as EDC, leading to a temporal control over charge density.^27^

Among the many systems introduced over the last decades, some have attracted considerable attention due to their versatile applications. One of such systems is the click-declick cycle of Anslyn and coworkers.^24^ Here, a Meldrum's acid derivative (MAD) is used to selectively capture one equivalent of thiol and one equivalent of amine (Scheme 1, left). This strategy can be used to functionalise peptides,^24^ or form cyclic peptides.^25^ Adding the thiol DTT can fully revert this reaction and release the initial thiol and amine. This system is signal-induced because the forward and backward process are two distinct steps for which the reagents are added subsequently. This is due to the cross-reactivity of MAD and DTT which prevents autonomous operation of this CRN. In another instance, Caddick, Baker, and coworkers show the reversible functionalisation of biomolecules such as proteins with a dibromomaleimide (DBM) (Scheme 1, centre).^26^ Unlike the typical thiol–maleimide bond, this formation is fully reversible in the presence of a second thiol which makes this biofunctionalisation especially attractive. One of the downsides is that to fully push the reaction back to the starting point, a large excess of second thiol is necessary. Contrasting these signal-induced CRNs, a prime example of autonomous CRNs are the EDC-driven systems pioneered by Boekhoven and colleagues,^27^ as well as the group of C. Scott Hartley.^28^ In Boekhoven's system, a glutamic or aspartic acid functionalised species can transiently be transformed into an uncharged anhydride with a carbodiimide such as EDC (Scheme 1, right). This anhydride will then spontaneously hydrolyse back to the starting acid, allowing for temporal control over the charge density in the system. The fact that only one reagent addition is necessary to fully cycle this system through an activated state B back to state A makes it autonomous. One of the downsides however is the poor control over the kinetics of the hydrolysis step.

Despite these promising works, CRNs controlled entirely through chemical signals remain relatively few, and still suffer from some common drawbacks such as waste accumulation and cross-reactivity.^29–33^ Furthermore, the poor control over hydrolysis reactions as part of a CRN, common in the deactivation step of many CRN systems,^27,30,34–38^ is one of the drawbacks that can be resolved in systems applying distinct reagents as stimuli instead of relying on hydrolysis for the recovery of the starting compound. New chemical reactions are needed to design more robust, versatile and efficient CRNs that can respond to chemical signals. Additionally, if we aim to develop CRNs that are compatible with living systems, a novel approach is needed to find chemistry that is less toxic than commonly used fuels such as carbodiimides, or methylating agents such as methyl iodide.^27,28,36,38,39^ We envisioned that conjugate additions could resolve many of these challenges and present a valuable extension of the systems chemists’ toolbox for developing CRNs to enable new applications in material science, drug delivery, as well as fundamental science to study in depth how CRNs function and how we can bridge the gap between systems chemistry and much more complex, biological systems.

### Versatility and drawbacks of conjugate additions

Conjugate additions are of immense importance in the formation of carbon–carbon and carbon–heteroatom bonds. They have been investigated in depth over the last 150 years and comprise a large range of reactions that are indispensable in modern organic chemistry.^40^ Among the large range of additions of different nucleophiles to different acceptors,^40–43^ many of which can also be run with high enantioselectivity,^40,44–46^ some of the key transformations are the Michael addition to form carbon–carbon bonds,^47^ the Robinson annulation which is one of the key methods to close rings,^48^ as well as the thiol-, aza-, and phospha-Michael additions, which enable the formation of carbon–sulfur, carbon–amine, and carbon–phosphorus bonds respectively, commonly applied in material science^49–51^ and protein functionalisations.^52–58^ Furthermore, nucleophilic conjugate additions are of biological relevance as the involved Michael acceptors possess high bioactivity due to their reactivity towards various nucleophiles, making them good candidates as covalent modifiers,^59^ as well as warheads in activity-based probes.^60^ Although their sometimes indiscriminate reactivity towards nucleophiles is responsible for the carcinogenicity and toxicity of some Michael acceptors,^61^ precise tuning of their reactivity and substituents to precisely fit the pocket of the active sites of a biological target are commonly applied in the process of drug discovery.^61,62^ Furthermore, cytotoxicity of even small-molecule Michael acceptors strongly depends on their structure, making biomedical applications possible depending on the choice of molecules.^63^

**Scheme 2:**
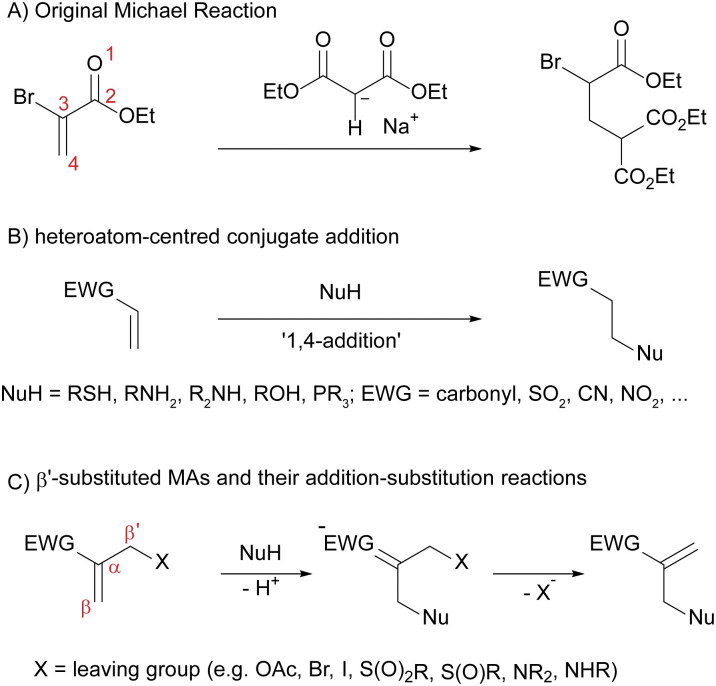
(A) The original Michael reaction, discovered by A. Michael, comprises the addition of a malonate to a stabilised acrylate ester. (B) The 1,4-addition of heteroatom-centred nucleophiles to Michael acceptors, which is of central importance to this work. (C) β′-Substituted Michael acceptors, which can undergo addition–substitution reactions, maintaining the active double bond moiety.

Conjugate additions always involve the attack of a carbon- or heteroatom-centred nucleophile, *i.e.* Michael donor, on an electron-poor, unsaturated electrophile, called the Michael acceptor (Scheme 2A and B). These Michael acceptors are a wide class of compounds that consist of an electron-withdrawing group such as carbonyls, nitriles or sulfones in conjugation with one or more double or triple bonds. This wide substrate scope makes these reactions especially attractive in synthetic organic chemistry. There are several ways that nucleophiles can add to these Michael acceptors: in a 1,2-addition reaction which is especially relevant for carbonyls, in a 1,4-addition reaction which will be of central importance for this work (Scheme 2B), and—in larger conjugated systems—also in a 1, (2*n* + 2)-addition reaction (*n* > 1). Upon searching the literature for these reactions, it becomes clear that the terms ‘conjugate addition’, ‘Michael addition’ and ‘Michael reaction’ are often used interchangeably. Strictly speaking however, only the addition of carbon-centred nucleophiles to Michael acceptors is considered as a Michael addition, whereas the addition of stabilised carbon-centred nucleophiles is termed Michael reaction (Scheme 2A). ‘Conjugate addition’ describes all such addition reactions of any nucleophile to an unsaturated, conjugated electrophile. Hence, we will refer to the addition reactions of heteroatom-centred nucleophiles to Michael acceptors (Scheme 2B) simply as ‘conjugate additions’ throughout this article.

Despite the chemical versatility and often high yields and short reactions times, conjugate additions suffer from some drawbacks. Most notably, they are often difficult to reverse and the Michael-accepting functionality gets lost during the addition reaction, with some exceptions such as the Robinson annulation where the elimination of water follows the addition and ring closure steps, or the Morita–Baylis–Hillman reaction where the elimination of the catalyst regenerates the double bond, among others.^48,64^ This loss of functionality is especially problematic if the Michael-accepting moiety is desirable for further addition- and substitution-reactions such as late-stage functionalisation procedures.

### β′-Substituted Michael acceptors: addition–elimination reactions

To overcome this problem, the group of R. Lawton developed a special class of Michael acceptors that carry a leaving group in the β′-position (Scheme 2C).^65^ They are also commonly called α-substituted MAs,^66,67^ however we will refer to them as β′-substituted to put emphasis on the presence of a leaving group in this position and distinguish them from other α-substituted species that cannot undergo the same reactions. Although these compounds also undergo the typical 1,4-addition reactions, the leaving group in β′-position enables an elimination reaction to take place upon conjugate addition of the nucleophile. This creates a new Michael-accepting double bond moiety (Scheme 2C). This means that the MA moiety is retained in the molecule and is available for further conjugate addition–elimination reactions. In classical conjugate additions (Scheme 2A and B), neither is the MA moiety retained, nor is the reaction reversible under such mild conditions.^40^ For a second conjugate addition–elimination reaction to occur quantitatively on β′-substituted MAs, it is essential that the attacking nucleophile is stronger than the previous one, to enable the elimination of the first nucleophile as a new leaving group.^68^ Furthermore, the feature of retaining the double bond upon elimination allows for a dynamic exchange of nucleophile and leaving group on the β′-substituted MAs. This is why they are commonly referred to as ‘equilibrium transfer alkylating crosslink’ reagents (ETACs).^58,69^ Due to these favourable properties, β′-substituted MAs have since been applied as molecular yardsticks,^69^ as tools for biofunctionalisations,^54,57,58,70–73^ in chemical switches,^68^ as well as in the random walk of small molecules.^74^

**Scheme 3:**
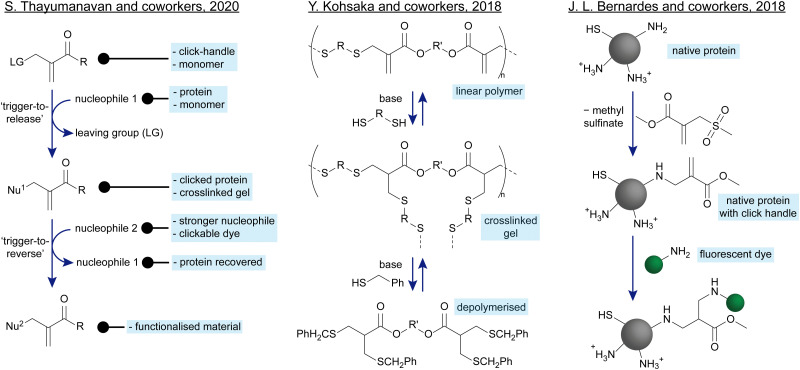
Recent applications of β′-substituted MAs. Left: S. Thayumanavan and coworkers have performed in-depth kinetic studies on the addition–elimination reaction of these species and demonstrated their applications for crosslinkable gels, and as click handles.^68^ Centre: Y. Kohsaka and coworkers have shown different applications of β′-substituted MAs in polymer materials. One of them involves the fully reversible crosslinking of MA-based materials.^75^ Right: J. L. Bernardes and coworkers have demonstrated the chemoselective modification of native proteins on the most nucleophilic lysine residue(s) with a β′-substituted MA as a reactive click handle.^72^

Of special note is the work of S. Thayumanavan and coworkers (Scheme 3, left). They have studied in depth the impact that different substituents have on the kinetics of addition–substitution reactions. Further, they show that β′-substituted MAs can be used both as click handles for biofunctionalisations as well as monomers to make reversibly crosslinked networks.^68^ Kohsaka and coworkers have performed several in depth studies on how to use these MAs in materials as well. In one of their works (Scheme 3, centre), they show how a linear polymer made of bisfunctional MAs and bis-thiols can be crosslinked with additional bis-thiols. They then demonstrate the fully reversible, dynamic nature of these thiol-MA adducts by adding an excess of monofunctional thiol which leads to chain scission in the presence of catalytic base, making the polymer fully degradable in response to a chemical signal.^75^ In another example, J. L. Bernardes and coworkers have demonstrated the chemoselectivity of β′-substituted MAs (Scheme 3, right). By adding a MA with a sulfinate leaving group, they show a selective modification of the most nucleophilic lysine of a native protein, leaving cysteine and less nucleophilic lysines untouched. This work offers a strategy to chemoselectively introduce a clickable handle onto native proteins, which can further be functionalised with a fluorescent dye.^72^

Despite their versatile applications, β′-substituted MAs also suffer from some downsides, such as a loss of double bond functionality upon double additions.^76^ Furthermore, the reactivity and high reversibility of these species may lead to a decreased stability of the desired products.^58,76^ Lastly, double bonds typically tend to be relatively labile groups and may degrade over time thermally, in light, or in the presence of radical sources, making the addition of inhibitors necessary for prolonged storage.^77,78^

Besides these findings on β′-substituted MAs, significant work has been undertaken on α- and β-substituted MAs as well.^66,67,75,79–82^ On the one hand, MAs with a leaving group in α-position cannot undergo subsequent addition–elimination reactions. β-Substituted MAs on the other hand can react similarly to β′-substituted MAs. One famous example of β-substituted MAs are Meldrum's acid derivatives developed by the group of Eric Anslyn mentioned above (Scheme 1, left),^24^ commonly applied in the formation of cyclic peptides, as well as click–declick plastics.^24,25,83^

However, due to the steric hindrance that leaving groups in β-position present towards incoming nucleophiles, we decided to start working with the β′-substituted analogues. We sought to use their interesting features to derive new out-of-equilibrium fuel-driven CRNs that can have potential applications in a wide range of fields.

**Fig. 2:**
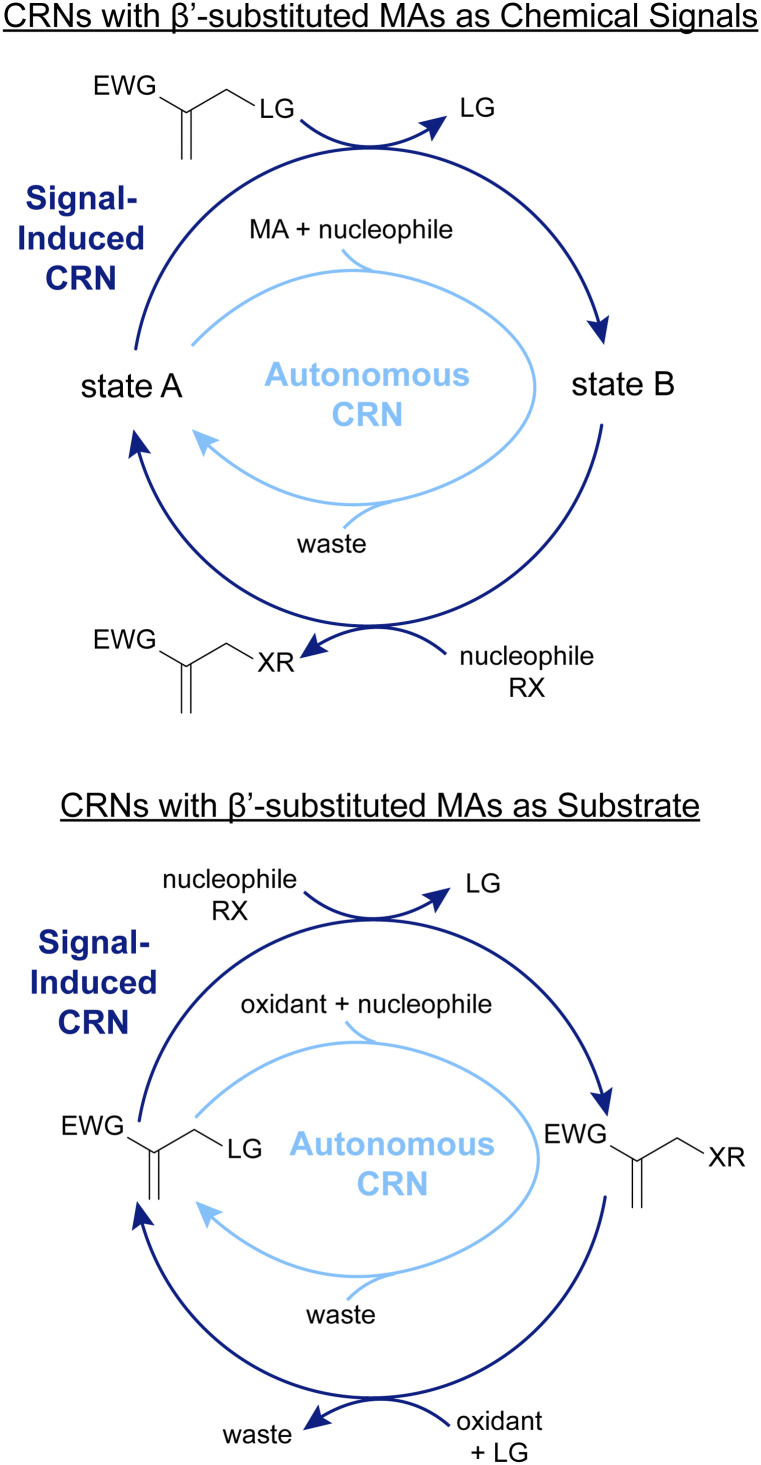
Signal-induced and autonomous CRNs in which the β′-substituted MAs are used as (A) chemical signals, and (B) substrates. The different properties of state A and state B lead to various applications of these CRNs explained in detail below.

The following sections will showcase, in more detail, the progress we have made in applying this chemistry in the context of CRNs (Fig. 2). We have grouped our works by area of application. Fig. 2 also gives a comprehensive summary of the nature of the systems we present: they can be classified by whether the β′-substituted MA is the chemical signal (top), or the substate (bottom) of the CRN. Furthermore, a dark-blue colour indicates a signal-induced CRN, whereas light-blue colour indicates an autonomous CRN. We will start by giving a brief overview of how we conceived some of the ideas and concepts for these projects and what we learned about the reactivity of the species involved along the way.

## MAs as a basis for CRN chemistry and their reactivity

**Scheme 4:**
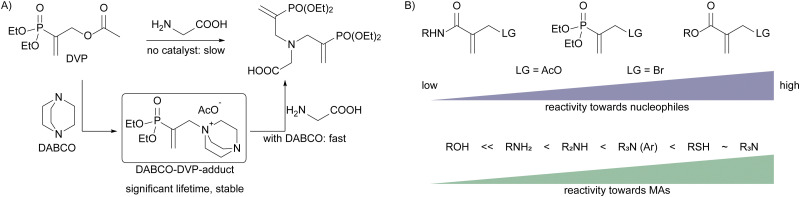
(A) Diethyl(α-acetoxymethyl)vinylphosphonate (DVP) reacts slowly with glycine to form a double adduct of DVP on glycine. In the presence of DABCO, this reaction proceeds roughly 15-fold faster *via* a DABCO-DVP adduct, which possesses a significant lifetime and can be isolated. (B) Depending on the electron-withdrawing group (EWG) and leaving group (LG), MAs possess vastly different reactivity towards nucleophiles, just as different nucleophiles show different reactivity towards MAs. These trends can act as a general guideline for the reader.

We initially looked at β′-substituted MAs through the lens of catalysis and host–guest chemistry. We studied how host–guest chemistry can be used to encapsulate 1,4-diazabicyclo(2.2.2)octane (DABCO), which catalyses the addition–substitution reaction of nucleophiles on β′-substituted vinylphosphonates, and hence tune catalytic rates.^84^ β′-Substituted vinylphosphonates and their reactions with nucleophiles have been studied previously,^85,86^ and we found that cucurbit[7]uril (CB[7]) can indeed be used to tune the organocatalytic activity of DABCO.^84^ Yet, what appeared in these experiments was that DABCO is a mediocre catalyst at best. We regularly observed a build-up of the catalytic DABCO-MA intermediate (Scheme 4A), which could even be isolated, due to its relatively low reactivity. Although these amine-MA adducts, their reactions, and applications in synthesis have been studied previously,^87–89^ they have not been applied in the context of CRNs before. As these species are charged, have a significant lifetime and can react with other nucleophiles to recover the initial, uncharged amine species, we hypothesised that such catalytic intermediates could be excellent candidates as substrates in a CRN which can switch between uncharged (state A, Fig. 1 and 2A) and charged (state B, Fig. 1 and 2A) species in response to a chemical signal. This finding, that β′-substituted MAs can be used to cycle tertiary amines between an uncharged and charged state reversibly, became the starting point for the projects outlined below.

These early experiments with β′-substituted MAs also revealed some general trends regarding stability and reactivity that became useful for future projects (Scheme 4B). Along those lines, extensive studies from the group of S. Thayumanavan, as well as in our lab, showed that both the electron-withdrawing group (EWG) and leaving group on the MA, as well as the characteristics of the nucleophile strongly impact the overall kinetics of the reactions and stability of the involved species.^13,63,68,76,84,90^ While allylic amides were relatively unreactive, diethyl(α-acetoxymethyl)vinylphosphonate (DVP) was found to have intermediate reactivity and allylic esters were found to be most reactive, with the acetate leaving group leading to slower addition-substitution reactions than the bromide leaving group (Scheme 4B, top). Among nucleophiles, we found aliphatic tertiary amines to react fastest, at comparable rates to thiols, which react faster than aromatic tertiary amines, secondary amines, primary amines, and lastly alcohols, which do not show any meaningful reactivity with β′-substituted MAs (Scheme 4B, bottom).

## MA-based CRNs for material applications

Inspired by the findings regarding lifetime and stability of DABCO-MA adducts, we proceeded to explore potential material applications for this new chemistry. While activation of building blocks to form transient materials with a chemical signal is common, deactivation reactions are most commonly driven by hydrolysis or pH changes.^30,32,35^ We envisioned that the chemistry of β′-substituted MAs could be another valuable asset in the systems chemist's toolbox to chemically control both activation and deactivation reactions in a CRN coupled to a material's properties.

**Fig. 3:**
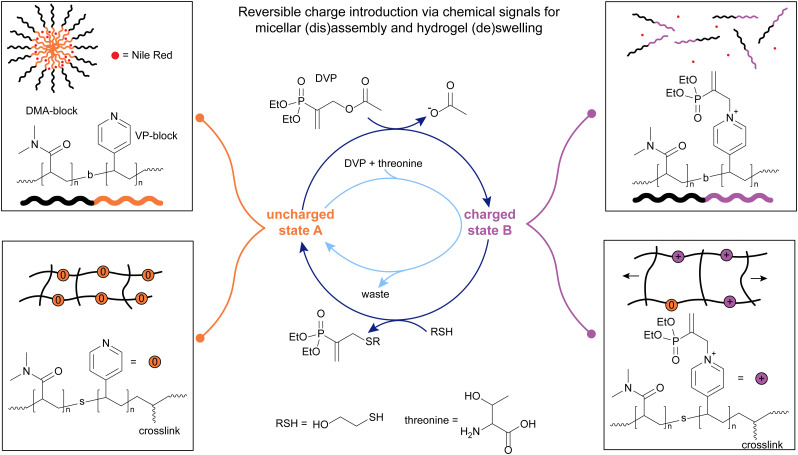
The addition of DVP on pyridine was applied to DMA-VP block copolymers (top left, uncharged state A) to trigger the disassembly of micelles encapsulating Nile red (top left). A nucleophile (thiol or amine) was applied to switch from the disassembled charged state (top right, charged state B) back to state A. Also, this chemistry was applied to crosslinked, DMA-VP statistical copolymers (bottom left) to switch between a swollen (bottom right) and unswollen (bottom left) gel state. The letter b denotes a block-, the letter s a statistical copolymer. Experiments typically conducted in pH 7.4 phosphate buffer (0.1 M for signal-induced, 0.5 M for autonomous CRN).

Starting from small molecule studies, we decided to work with DVP and tertiary amines. We found that apart from DABCO, pyridine is another especially suitable amine to react with DVP to yield a relatively stable, charged pyridine-DVP adduct, that can further react with other nucleophiles to recover the initial, uncharged pyridine species (Fig. 3).^13^ Furthermore, we found that strong nucleophiles, *i.e.* thiols, react very fast and with high yields with charged amine-DVP adducts, recovering the uncharged amine and forming a thiol-DVP adduct as waste. Mixing DVP with a substoichiometric amount of amine and adding the same amount of thiol in four portions led to a repeated cycling of the amine between its charged adduct and its free, uncharged state. Hence, tertiary amines such as DABCO and pyridine could be activated to yield a charged state through the addition of DVP and deactivated again to the uncharged state through addition of a thiol in a signal-induced CRN. Weaker nucleophiles such as threonine, a primary amine, react with the amine-DVP adduct much more slowly than thiols, and with somewhat lower yields, allowing for control over the lifetime of the activated species. This is due to the relatively high p*K*_a_ (9.1) of threonine,^13^ which means that the majority is in its protonated state at physiological pH. Mixing DVP, pyridine and threonine, we observed an autonomous operation of the CRN, with a transient formation of the charged pyridine-DVP adduct. Varying the ratios between chemical signal, pyridine and threonine allowed for a control over the amplitude of the activation step.^13^

With the chemistry in hand for a new CRN that can switch between an uncharged state A and a charged state B, we applied these reactions on the material scale. We chose to apply this chemistry to micelles, which have been previously shown to undergo oscillatory behaviour and function as nanoreactors or stimuli-responsive drug delivery systems, in response to chemical signals within CRNs.^5–8^ DMA-VP-block copolymers were used to make amphiphilic micelles, confirmed *via* DLS and TEM. *N*,*N*-Dimethylacrylamide (DMA) was used as the water-soluble block, while 4-vinylpyridine (VP) was used as the active block that can switch between a charged (Fig. 3, top right) and uncharged state (Fig. 3, top left). Vinylpyridine is especially attractive due to its p*K*_a_ of 5.0 ± 0.3, meaning that it is uncharged at physiological pH, making it the hydrophobic block in its non-activated state.^13^ To probe the signal-induced (dis)assembly of the micelles, an excess of DVP was added to the micelle solutions. We found the micelles to degrade fully, due to the formation of charges in the hydrophobic block *via* reaction of pyridine units with DVP, within approx. 105 hours. Addition of thiol very swiftly (approx. 1 hour) leads to the reformation of the micelles, which then degraded again due to the presence of an excess of DVP. We repeated the disassembly-assembly cycle 4 times, until all of the DVP had depleted. ^1^H-NMR studies revealed that even relatively low conversions of pyridine units to their charged pyridine-DVP adducts (approx. 28%) are sufficient to induce micellar disassembly due to charge repulsion.^13^

Next, we tested whether loaded micelles can undergo the same (dis)assembly steps, leading to the release and re-uptake of a cargo. For this, Nile red (NR) dye was chosen, due to its strong fluorescence in hydrophobic environments, which is quenched in water.^91^ We managed to reproduce the behaviour found for the unloaded micelles: The loaded micelles released 95% of NR within 54 hours after introducing DVP, due to micellar disassembly, whereas fluorescence increases rapidly, within 4 hours, upon introduction of a thiol, due to re-assembly of the micelles and incorporation of NR into the hydrophobic core (Fig. 3, top left and right). Although repeated thiol additions led to repeated (dis)assembly cycles, the dampening of the fluorescence response was stronger than conversion-dampening in unloaded micelles, due to waste accumulation in the hydrophobic micelle blocks, leading to less NR uptake over several additions of DVP. Similar to the unloaded micelles, we tried to achieve a transient NR release with threonine as a nucleophile, instead of a thiol. Indeed, upon simultaneous addition of DVP and threonine to the loaded micelles, 83% cargo were released over the following 19 hours, upon which fluorescence started to increase again, indicating cargo-reuptake to near original levels over the following 19 days.^13^

In addition to the micellar system, we became interested in the (de)swelling of crosslinked hydrogels in response to the reversible introduction of a charge with this MA chemistry (Fig. 3, bottom left and right). For this application, we synthesised statistical DMA-VP copolymers with blocks of bis(acrylamide) crosslinkers. These block-shaped gels swell in aqueous conditions (90 wt% water), due to the presence of the hydrophilic DMA units. Upon introduction of equimolar amounts of DVP, relative to pyridine units, a size increase of 106 ± 16% was observed over 96 hours, due to the introduction of charges in the network, leading to increased water uptake and charge repulsion. Upon addition of a thiol, deswelling was observed over a similar timescale, back to roughly the initial size of the gels, demonstrating the time-programmed deswelling of crosslinked hydrogels. Similarly, to showcase an autonomous (de)swelling behaviour, we introduced 2.1 eq. DVP and 8.0 eq. threonine simultaneously. Over 168 hours, the gels expanded by 80 ± 11%, and reached initial dimensions after 504 hours.^13^

Concluding, these results show the successful applications of β′-substituted MAs for both the time-programmed, and autonomous introduction and removal of charges on tertiary amines in response to chemical signals. These findings were applied both to the (dis)assembly of micelles, showcasing the release and re-uptake of NR as a model cargo, as well as the (de)swelling of crosslinked hydrogels.

**Fig. 4:**
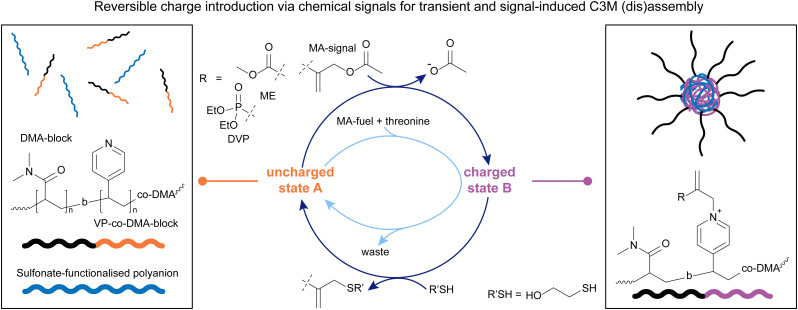
2-(Acetoxymethyl)acrylate (ME) and DVP were applied to solution of DMA-VP block copolymers with sulfonate-functionalised polyanions (poly(sodium 4-styrenesulfonate), PSS) (left, uncharged state A) to form complex coacervate core micelles (C3Ms, right, charged state B). A nucleophile was applied to switch back to state A, both in a signal-induced and autonomous manner. The letter b denotes a block-copolymer. Experiments typically conducted in pH 7.4 phosphate buffer (0.1 M for signal-induced, 0.25 M for autonomous CRN).

With a system in hand that offers precise and fully reversible control over charge introduction on tertiary amines, we decided to look at complex coacervate core micelles (C3M) as another interesting area in which to apply the chemistry of β′-substituted MAs (Fig. 4).^90^ C3Ms form *via* coacervation of positively and negatively charged polyelectrolytes, with neutral, water-soluble blocks attached to at least one of those polyelectrolytes.^9–12^ Unlike traditional micelles, the hydrated, water-insoluble cores of C3Ms are capable of encapsulating, protecting, and releasing complex biomolecules for applications in stabilised dispersions, and therapeutic delivery systems, among others.^92–94^ Although C3Ms have been extensively studied in the past,^10^ precise control over their lifetime in response to chemical signals at physiological pH has not been achieved previously. We decided to work with a VP-*co*-DMA-*b*-DMA polymer (Fig. 4) and apply the same addition–substitution chemistry we previously explored,^13,84^ in the presence of polyanionic species. We decided to incorporate DMA units into the VP block as well, to avoid micelle formation in the uncharged state (Fig. 4 left), as observed in the previous project.^13^ This process starts by combining the uncharged polymer with a polyanion (in this case PSS, poly(sodium 4-styrenesulfonate), yielding a homogeneous polymer solution). Upon introducing positive charges on the polymer through addition of a β′-substituted MA (DVP or ME), the charged copolymer can then form C3Ms with the polyanion in solution (Fig. 4 right). The addition of different nucleophiles, *i.e.* a thiol or amine, can revert the C3Ms back to the disassembled state, in a signal-induced or an autonomous manner (Fig. 4).^90^

Mixing the neutral polymer with one equivalent of MA and a sulfonate-functionalised polyanion in buffer at physiological pH led to the formation of the charged polymer (Fig. 4, charged state B). For this system, we studied two MA species, DVP and 2-(acetoxymethyl)acrylate (ME) (Fig. 4), with different electrophilicities. With DVP, 65% of the VP units were functionalised after 120 hours, whereas ME reacted with 80% of VP units in just 5 hours. This shows that the reactivity of the chemical signal can be used to tune the kinetics of the forward reaction, introducing charges on the polymer. Upon this functionalisation, these block copolymers form C3Ms by complexation with the polyanion, which was confirmed *via* DLS and TEM. To study the signal-induced disassembly of these C3Ms, a thiol was added. This resulted in the swift recovery of the uncharged starting polymer within 5 minutes, with generation of the thiol-functionalised waste. This system was also run under physiological conditions at 37.5 °C. Furthermore, we found that both systems can be cycled between a solution and an assembled C3M state at least twice.^90^

Upon establishing that a signal-induced assembly and disassembly of C3Ms is possible by adding 1.0 eq. of MA-signal (assembly) and 1.0 eq. of a thiol (disassembly), we investigated the transient disassembly of C3Ms by adding an excess of MA-signal. Indeed, in the presence of 3.0 eq. of MA-signal, the addition of 1.0 eq. thiol resulted in the temporary disassembly of the C3Ms. Subsequently, the polymers autonomously returned to an assembled C3M state in 2 hours in the presence of ME. When DVP was used instead, this process took 193 hours. The cross-reactivity between thiol and MA species was minimal in both cases due to the preferred reactivity with the pyridine-MA adduct over the MA-signal, demonstrating that this system can also be run autonomously. *Vice versa*, we found that adding an excess of a weak nucleophile, in this case threonine, with which the deionisation reaction is slower than the ionisation step, can lead to the transient assembly of C3Ms upon addition of small portions of MA. Again, DVP led to a more long-lived transient assembly of C3Ms than ME, allowing autonomous time control over the assembled regime.

**Fig. 5:**
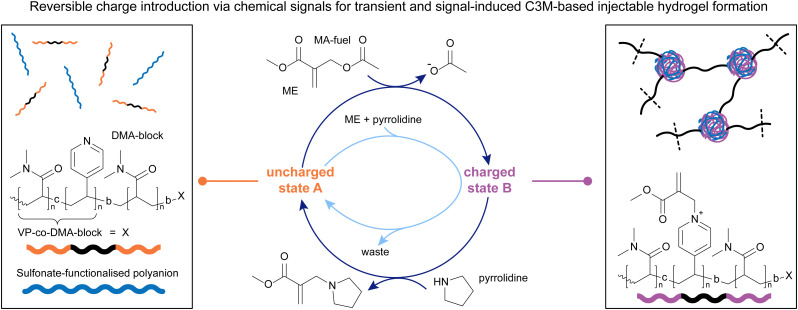
ME was applied to a solution of VP-DMA-*block*-DMA-*block*-VP-DMA triblock copolymers and sulfonate-functionalised polyanions (PAMPS_236_, poly(sodium 2-acrylamido-2-methylpropane sulfonate)) (left, uncharged state A) to yield a C3M-based, injectable, crosslinked hydrogel (right, charged state B). Pyrrolidine was applied, both in a signal-induced and autonomous manner, to return to the uncharged solution state A. The letter c denotes a copolymer, the letter b denotes a block-copolymer. Experiments typically conducted in 0.1–0.15 M pH 7.4 phosphate buffer.

After studying how β′-substituted MAs with different residues can be used in combination with nucleophiles of different strength to achieve the signal-induced, as well as autonomous assembly and disassembly of C3Ms, we decided to apply this knowledge to create injectable, coacervate polymer hydrogels (Fig. 5). In this more recent work, we functionalised a DMA-polymer with VP-DMA-blocks on both ends. These reactive ends enable the formation of C3Ms in the presence of polyanions (in this case PAMPS_236_, poly(sodium 2-acrylamido-2-methylpropane sulfonate)), upon application of an MA, acting as crosslinks.^63^ Although CRN-based control of coacervation has previously been shown and applied in different fields,^95–97^ this is the first example that demonstrates chemically induced assembly and disassembly of C3Ms (Fig. 5 right and left, respectively) under physiological conditions on a macroscopic scale, *i.e.* a gel.

Similar to previous works, we found the triblock copolymer (Fig. 5, left) to react with 1.0 eq. of ME to about 80% conversion over 5 hours, with higher conversions achievable with an excess of ME. The deactivation step with pyrrolidine proceeded within five hours to yield approx. 90% uncharged state A. Combining the triblock copolymers with a polyanion and adding 1.0 eq. of ME led to the formation of a macroscopic gel within 30 minutes, and a return to solution state within 5 minutes of adding pyrrolidine. We managed to cycle between the gel and sol states three times with subsequent additions of ME and pyrrolidine, and furthermore demonstrated an autonomous behaviour upon adding an excess of ME, followed by stoichiometric additions of pyrrolidine (Fig. 5, central, light blue cycle). Additionally, adding larger amounts of ME leads both to stronger gels, as well as shorter gelation times (between 30 and 90 minutes), enabling a degree of control over gel properties by tuning the amount of chemical signal applied to the system, as well as the initial concentration of the triblock copolymer. Rheology measurements showed that the gels can also be destroyed with high strain (>200%), rapidly recovering to their initial strength upon lowering the strain back to 5%, demonstrating the self-healing nature of these materials. This inspired us to apply these materials as injectable hydrogels. Indeed, we found that due to their destruction under high shear forces and rapid self-healing, the gels could be injected through needles ranging from 20G down to 26G (0.9–0.45 mm), making these materials good candidates for biomedical applications, such as drug delivery. Another important criterion is the degradability of the injected material under physiological conditions. We show that the gels easily degrade in cell culture medium at 37 °C within 2 hours, whereas their degradation in phosphate buffer is much slower (approx. 24 hours). Although their cytotoxicity is relatively high due to the presence of ME, we found that substituting ME with DVP resulted in a significantly lower cytotoxicity of the gel system.^63^

In a follow-up work, we wanted to further demonstrate the relevance of this chemistry in cellular environments due to the low cytotoxicity of DVP. We became interested in looking at how reversible cationisation can be used to encapsulate plasmid DNA (pDNA) and shuttle it into cells, leading to enhanced gene expression. This work used polymers that had been cationised by reaction with a MA prior to complexation with DNA. Unlike the other results presented in this Feature Article, this work does not apply MA chemistry in the scope of a full CRN. The backward process, *i.e.* to decationisation of charged pyridine units, was used to selectively release pDNA in cells.^98^ The core idea of this project is based on the relatively low concentration of nucleophiles in extracellular medium, whereas the concentration of amino acids and thiols, specifically glutathione, is much higher in intracellular medium, leading to the release of pDNA after transfer into the cell. We show in model experiments that pDNA can be released from the cationic polymer DNA complexes (polyplexes) within 4 hours in cell culture medium and within 1 hour in the presence of 1 mM glutathione.^98^ Permanently cationised control polyplexes do not lead to a pDNA release in the presence of nucleophiles. Further, a range of micelles, polyplexes and lipopolyplexes were prepared. We found that the lipopolyplexes transfect cells and demonstrated with control experiments that decationisation in the cellular medium is responsible for the enhanced gene expression. Besides these findings, this work also shows the significantly reduced cytotoxicity of the reversibly cationised polyplexes, as well as the cationization reagent DVP, compared to permanent cations and other commonly applied reactants often applied in CRNs.^98^ These results extend the scope of applications of MA chemistry beyond material applications into the biological realm, overcoming some of the previous challenges such as high cytotoxicity.

## MA-based CRNs for signal amplification

**Fig. 6:**
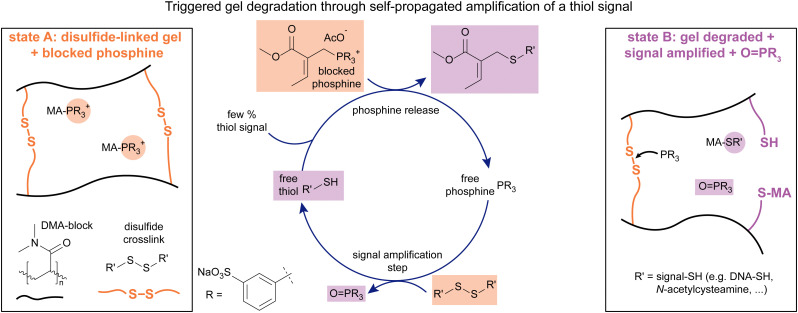
Blocked phosphine was applied in a crosslinked disulfide-based gel (state A). The addition of a thiol signal (the analyte) leads to the amplified release of phosphine and more thiol, degrading the network over time (state B), both in response to a chemical (thiol addition) and a mechanical signal (force-generated thiyl radicals as a thiol). Experiments typically conducted in 0.1 M pH 7.6 phosphate buffer.

In more recent work, we looked at different applications beyond the material scope and focused on signal amplification. The amplification of chemical signals has many promising applications,^99–101^ but systems driven only by simple, chemical reactions remain few and often some downsides remain such as high background reactivity or low sensitivity.^99,102–106^ We chose to use β′-substituted MAs for the protection of phosphines (PR_3_), in the form of phosphonium salts. These salts can respond to a chemical signal (thiol), yielding a free phosphine which can reduce disulfides to the corresponding free thiols (Fig. 6, see also Notes for explanation of thiol-adduct structure††The structure of the thiol-MA adduct in Fig. 6 may suggest a direct substitution of the phosphine on the β-carbon instead of on the β′-carbon like in the other projects. This is however not the case. The thiol attacks adjacent to the methyl group. As the product is dynamic however, it will slowly equilibrate to the thermodynamically more stable product depicted in Fig. 6 in the presence of free thiols. For more details, see the Supplementary Information of the cited work.).^107^ Due to the stoichiometry of the reduction of disulfides with phosphines, each phosphine can liberate two thiol molecules, of which one can in turn release a new phosphine, making this cycle self-propagating, amplifying the initial thiol signal and producing a thiol-functionalised MA and a phosphine oxide as waste species.

Kinetic studies on the system containing blocked phosphine and disulfide (Fig. 6, state A), supported by modelling the kinetics, proved the concept and revealed the influence that the ratios of reagents have on the reaction progress.^107^ The reaction was primarily tracked studying the amount of free phosphine over time, which is the transient species that gets first released upon introducing the thiol signal, then consumed by reducing the disulfide. We found that adding more signal to the system leads to an earlier and more pronounced peak of released phosphine (PR_3_). Having more disulfide present in the system at the start leads to an earlier, but less pronounced peak of free phosphine, while starting with more blocked phosphine leads to a bigger accumulation of free phosphine over time. Having understood the role that the chemical composition of the system plays, we synthesised a dimethylacrylamide network gel crosslinked by disulfide bonds, containing blocked phosphine. We used this gel to test whether these networks can degrade in response to a thiol signal, *N*-acetyl cysteamine in this case. Indeed, upon introducing 5% of a thiol signal, gel degradation was observed within 168 hours, whereas the reference gel remained stable for over 13 days. Equally, for several biologically relevant thiols, glutathione, bovine serum albumin, and thiol functionalized DNA, gel degradation was also observed with sensitivities ranging down to 0.132 μM of thiol signal. Although lower signal concentrations (*e.g.* 0.001% thiol functionalized DNA *vs.* 1% glutathione) also led to slower kinetics, eventual degradation can still be observed at such low signal input. Finally, the impact of mechanical damage on the gels was investigated. We found that cutting the gels significantly shortens their time to full degradation. The results indicate that this is due to force-generated thiyl radicals which can abstract H-atoms from the solvent surroundings, generating a thiol signal *in situ*, or adding directly to the double bond of the blocked phosphine, generating a radical which can release the phosphine. This work demonstrates the potential of β′-substituted MAs to be applied in signal amplification and as sensors for both chemical and mechanical signals.

## MA-based CRNs for oxidation-driven pathway control and MA recovery

In all the previously described works, the purpose of the MA was to (transiently) introduce a positive charge in the system. Upon addition of a nucleophile, the MA moiety is then transformed into a waste product. We wondered whether we can also use these β′-substituted MAs as a substrate, maintaining the active MA moiety throughout the CRN and using it to affect product speciation, as well as maintain the active MA site for further modification reactions. Typically, these MAs can be applied in chemical switches,^68^ however reverting back to the original state is challenging. As evident from our previous works, the reactions with strong nucleophiles such as thiols lead to a thermodynamic sink and the reaction with weaker nucleophiles such as amines to recover starting compounds is not possible.

**Fig. 7:**
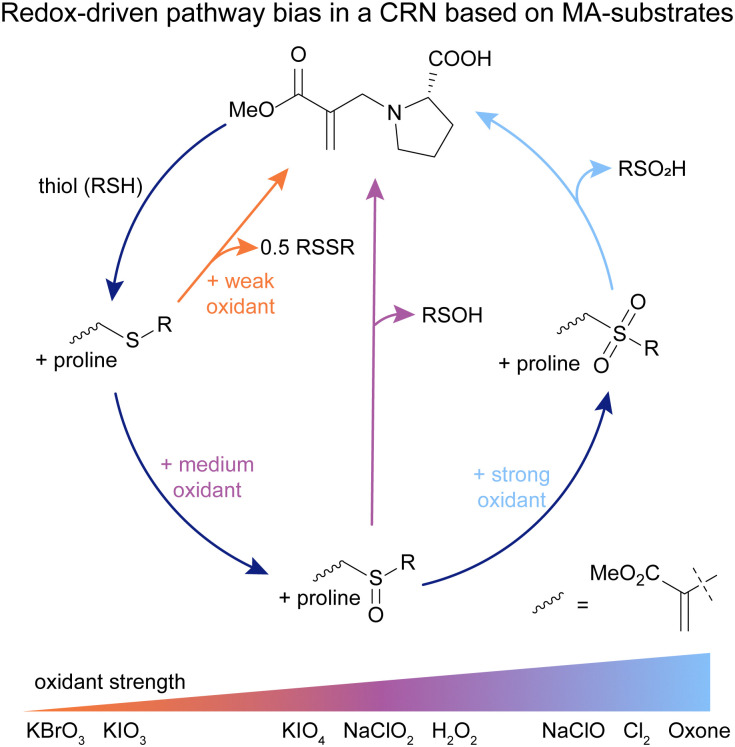
Proline-functionalised MA reacts with a thiol to form sulfide-MA. In the presence of strong oxidants, the sulfide-MA is activated towards nucleophilic attack *via* sulfone-MA, and proline-MA can be recovered (full cycle, light blue line). In the presence of weaker oxidants, two shunts open up, enabling the recovery of proline-MA *via* sulfoxide-MA (central pathway, purple line), as well as directly through sulfide-MA (left pathway, orange line), with different kinetics and side products compared to the full cycle. Experiments typically conducted in 9/1 0.5 M phosphate buffer (pH 7.0–8.0)/DMF.

Inspired by findings on protein functionalisations,^57,58,72,73^ and our previous efforts to design a CRN with MAs as substrates,^31^ we envisioned that oxidation chemistry can close the gap between β′-thiol-functionalised and β′-amine-functionalised MAs, by oxidising the thiol-functionalised species to a more electron-deficient MA, which enables the addition of an amine and recovery of the initial amine-MA (Fig. 7).^76^

Starting from exploring the separate steps of this network, we found that proline, as well as other primary and secondary amines, can recover the amine-functionalised MA from the sulfoxide- and sulfone-MAs with yields of 55 to 83%.^76^ Combining all reactions in a signal-induced cycle, we found that in the presence of the strong oxidant oxone, sulfone-MA forms and subsequently proline-MA recovers in a yield of 55% (Fig. 7, full cycle). This system can be run at least twice with similarly high recovery yields. Although this cycle works in a signal-induced manner, it cannot be run autonomously due to cross-reactivity of free thiol and oxidant. Even in the signal-induced sulfone cycle, we observed a range of side reactions such as oxidation of proline and proline-MA,^76^ which made us wonder whether some of these reactions can be mitigated by employing weaker oxidants. Studying medium-strength oxidants such as NaClO_2_, we found that indeed, proline-MA can also be recovered in yields of up to 83% with fewer oxidative side reactions occurring (Fig. 7, central pathway). Effectively, this creates a shunt to the full cycle in response the chemical potential of the oxidant, endowing the system with an adaptability similar to that found in shunts in nature, such as the glyoxylate shunt or cytochrome P450 shunt. To our surprise we found that even in the presence of weak oxidants such as KBrO_3_, the system also recovers proline-MA, not through oxidation of the sulfide-MA, but through oxidation of free thiol from the equilibrium between the sulfide- and proline-MA (Fig. 7, left pathway). Higher oxidation states and most of the side reaction found in the full cycle are completely bypassed in the presence of weak oxidants. Furthermore, the kinetics of the sulfide shunt are significantly slower than those found in the full cycle or the sulfoxide shunt.^76^

This CRN enables the use of β′-substituted MAs as the substrate and hence allows for their recovery. Further, we observed the presence of two different shunts to the full cycle in response to the strength of the employed oxidant. This leads to different CRN kinetics, side reaction profiles, and product speciation in response to a chemical stimulus, similar to shunts found in nature. These findings promise new applications for the exploration of CRN behaviour, transient materials and catalyst release.

## Conclusions

Chemical reaction networks (CRNs) are a great tool for systems chemists to study the complexity, adaptability, and responsiveness found in nature. They give insights into the underlying principles and open up possibilities to implement these principles in synthetic materials. Although previous works in this area show great progress and many promising applications as a result, a lack of signal-responsiveness, operation under non-physiological conditions, side reactions, and waste accumulation remain common issues in many CRNs. We have applied a special group of Michael acceptors (MAs), β′-substituted MAs, to develop two new types of CRNs. Using β′-substituted MAs as a chemical signal, the first type of CRN presents a highly versatile way to reversibly introduce charges on tertiary amines, both in a signal-induced and autonomous manner, for the application in chemical signal-based control over the behaviour of block copolymer micelles, C3Ms, hydrogels, and DNA polyplexes. Further, we show how this chemistry can also be used for signal amplification, demonstrating applications in naked-eye analyte detection. In another type of CRN, we apply these MAs as a substrate instead of a chemical signal, and show precise redox-based control over the pathway the CRN takes, its kinetics, and side reaction profile. We believe that this chemistry is extremely versatile and the works we have presented here are only the start of more exciting applications in different areas. Further, we believe that the reduced cytotoxicity of these species, specifically DVP, compared to other commonly used reagents in CRNs, can act as an entry into interactions of synthetic CRNs with living systems. We are currently working on exploring further projects in the fields of host–guest chemistry, where we have recently demonstrated application in control over supramolecular aggregate formation,^108^ triggered drug and catalyst release, transient materials, and beyond. We hope this overview can give researchers an understanding of the scope of applications and the reactivity of the involved species and aid in further discoveries based on novel CRNs that make use of this versatile chemistry.

## Author contributions

B. S. wrote the manuscript draft, designed the figures and conducted experiments and co-authored the draft in the work on oxidation-driven shunts. R. E. revised the manuscript, supervised the research described in this Feature Article, and secured funding for it.

## Abbreviations

C3M: Complex coacervate core micelle
CB[7]: Cucurbit[7]uril
CRN: Chemical reaction network
DABCO: Diazabicyclo(2.2.2)octane
DBM: Dibromomaleimide
DLS: Dynamic light scattering
DMA: *N*,*N*-Dimethylacrylamide
DMF: *N*,*N*-Dimethylformamide
DTT: Dithiothreitol
DVP: Diethyl(α-acetoxymethyl)vinylphosphonate
EDC: 1-Ethyl-3-(3-dimethylaminopropyl)carbodiimide
ETAC: Equilibrium transfer alkylating crosslink
EWG: Electron-withdrawing group
G (*e.g.* 20G): Needle gauge (Birmingham gauge)
LG: Leaving group
MA: Michael acceptor
MAD: Meldrum's acid derivative
ME: 2-(Acetoxymethyl)acrylate
NuH: General nucleophile (*e.g.* thiol)
PAMPS_236_: Poly(sodium 2-acrylamido-2-methylpropane sulfonate)
pDNA: Plasmid DNA
PSS: Poly(sodium 4-styrenesulfonate)
TEM: Transmission electron microscopy
VP: 4-Vinylpyridine

## Conflicts of interest

There are no conflicts to declare.

## Supplementary Material
